# Hypoxia and HIFs in Ewing sarcoma: new perspectives on a multi-facetted relationship

**DOI:** 10.1186/s12943-023-01750-w

**Published:** 2023-03-13

**Authors:** A. Katharina Ceranski, Martha J. Carreño-Gonzalez, Anna C. Ehlers, Maria Vittoria Colombo, Florencia Cidre-Aranaz, Thomas G. P. Grünewald

**Affiliations:** 1 Hopp-Children’s Cancer Center (KiTZ), Heidelberg, Germany; 2 Division of Translational Pediatric Sarcoma Research (B410), German Cancer Research Center (DKFZ), German Cancer Consortium (DKTK), Im Neuenheimer Feld 280, 69120 Heidelberg, Germany; 3 Regenerative Medicine Technologies Laboratory, Laboratories for Translational Research (LRT), Ente Ospedaliero Cantonale (EOC), Via F. Chiesa 5, CH-6500 Bellinzona, Switzerland; 4 Department of Surgery, Service of Orthopaedics and Traumatology, EOC, Lugano, Switzerland; 5 Department of Chemistry, Materials and Chemical Engineering “Giulio Natta”, Politecnico Di Milano, Via Mancinelli 7, 20131 Milan, Italy; 6 Institute of Pathology, Heidelberg University Hospital, Heidelberg, Germany

**Keywords:** Ewing sarcoma, hypoxia, HIF-1-a, HIF-1-b, ARNT

## Abstract

Hypoxia develops during the growth of solid tumors and influences tumoral activity in multiple ways. Low oxygen tension is also present in the bone microenvironment where Ewing sarcoma (EwS) – a highly aggressive pediatric cancer – mainly arises. Hypoxia inducible factor 1 subunit alpha (HIF-1-a) is the principal molecular mediator of the hypoxic response in cancer whereas EWSR1::FLI1 constitutes the oncogenic driver of EwS. Interaction of the two proteins has been shown in EwS. Although a growing body of studies investigated hypoxia and HIFs in EwS, their precise role for EwS pathophysiology is not clarified to date. This review summarizes and structures recent findings demonstrating that hypoxia and HIFs play a role in EwS at multiple levels. We propose to view hypoxia and HIFs as independent protagonists in the story of EwS and give a perspective on their potential clinical relevance as prognostic markers and therapeutic targets in EwS treatment.

## Introduction

Cancer is characterized on the one hand by irregular intracellular processes, and on the other hand by aberrant extracellular processes such as an altered interplay between cancerous cells and the tumor microenvironment (TME) [1–3]. One of the key aspects of the TME is hypoxia, which is generally defined as tissue oxygen concentration below the level needed for normal cell function [4]. Hypoxia will develop in most solid tumors because of increased cellular proliferation and oxygen need as well as of insufficient vessel formation and blood supply [5]. Studies identified the hypoxia inducible factor (HIF) protein family as key transcription factors that initiate the cellular adaptation to hypoxia [5–7]. To act as a transcription factor, the constitutively expressed subunit HIF-1-b and one of the three oxygen-dependently expressed subunits HIF-1-a/HIF-2-a/HIF-3-a must dimerize and bind to hypoxia response elements (HREs) in the target gene sequences [5, 6]. Thereby, HIFs regulate a multitude of functional pathways that can impact tumor activity, such as tumor vascularization via vascular endothelial growth factor (VEGF) [6], tumor metabolism via solute carrier family 2 member 1 (SLC2A1, better known as GLUT-1) [8] and Aldolase-C expression [9], and tumor motility and invasiveness via loss of E-cadherin and activation of Wnt/beta-catenin signaling [10, 11].

However, the role of HIFs in cancer cells goes beyond mediating the response to hypoxia: In fact, HIF-1-a can be upregulated through growth factors or oncogenic signaling cascades such as the phosphatidylinositol-4,5-bisphosphate 3-kinase (PI3K)/AKT serine/threonine kinase 1 (Akt) and Ras/Raf/mitogen-activated protein kinase (MAPK) pathway as well as through inactivation of tumor suppressors like the phosphatase and tensin homolog (PTEN) protein [12, 13]. This activation of HIF-1-a in normoxia through alternative pathways has been called pseudohypoxia [14] and opens a new perspective on HIF-1-a as a network hub to integrate other cellular and environmental signals beside hypoxia [13–15]. Furthermore, the regulatory mechanisms behind HIF-1-b (also named aryl hydrocarbon receptor nuclear translocator (ARNT)), HIF-1-a’s dimerization partner, have been suggested to be more complex, since HIF-1-b levels seemed to be influenced by hypoxia as well [16, 17]. Reversely, there is evidence for HIF-independent cellular responses to hypoxia, further challenging a simplistic view of hypoxia and HIF signaling [18]. Therefore, in this review, we intentionally do not use the terms hypoxia and HIF expression/signaling interchangeably but treat both factors separately. Furthermore, per definition, the terms *normoxia* and *hypoxia* are used in this review according to Hammond et al*.*, wherein normoxia refers to 21% oxygen tension, which is the atmospheric oxygen pressure and standard cell culture condition, and hypoxia refers to oxygen levels insufficient to meet the demand of the corresponding tissue [8]. Of note, the so called normoxic oxygen levels do not reflect the physiological oxygen tensions of most tissues, which vary between 3–7.4% oxygen (often referred to as *physoxia* [19]).

While the relevance of hypoxia in tumorigenesis and progression has been extensively studied and reviewed in many different cancer types [20–23], the current knowledge and particularities of hypoxia and HIF signaling in Ewing sarcoma (EwS) have not been systematically reviewed to date. EwS is the second most frequent bone-associated tumor predominantly occurring in children, adolescents, and young adults [24]. EwS was initially described more than 100 years ago by the American pathologist James Ewing in 1921, yet the precise cell of origin remains to be determined [24]. Despite this histogenetic uncertainty, EwS is genetically well characterized: In all cases, EwS is driven by chimeric transcription factors encoded by *FET::ETS* fusion oncogenes, most commonly Ewing sarcoma breakpoint region 1 protein (EWSR1)::Friend leukaemia integration 1 transcription factor (FLI1) (EWSR1::FLI1) (85% of cases) [24]. Hypoxia and HIFs are especially relevant in the context of EwS because: i) hypoxia is an integral component of the bone microenvironment playing an important role in the development of bone tumors [25–28]; ii) there is a direct interplay between HIF-1-a and EWSR1::FLI1 at the molecular level[29–31]; iii) there is a strong association of extensive tumor necrosis (likely caused by hypoxia) with metastasis and worse patient survival [32].

Thus, the aims of this review are to summarize the most recent findings on hypoxia and HIFs in the EwS context, and to provide a systematic coherence of the available data on this topic.

## The phenotype of EwS cells under hypoxia and/or HIF-1-a activity

### Proliferation

Several studies in EwS cell lines grown as monolayers (i.e., 2D) yielded controversial results concerning the effect of hypoxia on cellular proliferation [33–37]. However, Riffle et al*.* showed that in EwS spheroids, oxygen gradients divided cells according to distinct oxygen tension into populations with different proliferative states [4]. Specifically, EwS cells in the spheroid core stained for hypoxia and apoptosis markers but not for proliferation markers. Reversely, cells at the spheroid surface stained for Ki-67, indicating active proliferation, but exhibited neither hypoxia nor apoptosis markers [4]. Most interestingly, cells that resided at the interface between both populations and thus were exposed to moderate hypoxia were positive for Ki-67 staining and activated DNA damage repair (DDR) enzymes [4]. This suggests that cell cycle is compatible with moderate hypoxia but probably dependent on co-activated DDR [4]. In other tumor entities, such as head and neck squamous cell carcinomas, cells that retained proliferative capacity under hypoxia have been associated with lower survival and tumor aggressiveness, highlighting the clinical importance of studying these subpopulations [4, 38]. However, severe hypoxia is not compatible with EwS proliferation [4]. Regarding the influence of HIF-1-a on EwS cell proliferation, two studies conducted in normoxia and 1% oxygen condition showed that *HIF-1-a* silencing reduced proliferation of EwS cell lines in vitro, indicating a proliferation inducing effect of HIF-1-a in normoxia and hypoxia [31, 39]. However, Knowles et al*.* reported that knockdown of either *HIF-1-a *or* HIF-2-a *increased the proliferation of EwS cells under 0.1% oxygen tension, suggesting an anti-proliferative effect of both genes in EwS cells under very severe hypoxic conditions [36]. These discrepancies concerning the influence of *HIF-1-a*/*HIF-2-a* on EwS proliferation could be due to the different oxygen concentrations that were used in the experiments, implying that the influence of *HIF-1-a*/*HIF-2-a* on the EwS cell phenotype depends on the specific degree of hypoxia [19, 40]. Additionally, HIF-1-a levels vary exponentially within the range of hypoxic conditions, probably contributing to the above mentioned discrepancy of findings in EwS cells in hypoxia [19, 41]. In this context, several authors have emphasized the importance of monitoring pericellular oxygen levels and using standardized techniques for hypoxia models in vitro [41, 42]. This could reduce discrepancies in results and help to elucidate on the influence of hypoxia and HIFs on the EwS phenotype and pathophysiology.

### Apoptosis

Like in the case of cellular proliferation, diverse findings exist for the question on how hypoxia modulates apoptosis of EwS cell lines. Ryland et al*.* suggested that hypoxia does not induce apoptosis in EwS and found the epigenetic repression of the Potassium Voltage-Gated Channel Subfamily A Member 5 (KCNA5) gene to be involved in EwS cell survival under hypoxic stress [43]. Likewise, Kilic et al*.* confirmed reduced apoptosis of EwS cells under hypoxia and argued for a pro-survival role of hypoxia by showing that low oxygen tension protected EwS cells from chemotherapeutic-induced apoptosis [34]. However, other reports provided evidence that hypoxia activated apoptosis in EwS cell lines [36] and that hypoxia and apoptosis markers co-localized in the center of EwS spheroids [4]. In this context, it is intriguing that even studies that used the same cell line (A-673) and identical culture conditions (< 1% oxygen tension) yielded opposing results [34, 36]. On a similar note, the role of HIF-1-a in mediating apoptosis in EwS cells is controversial. Kilic et al*.* proposed that HIF-1-a protected EwS cells from apoptosis under hypoxia, as knockdown of *HIF-1-a* or therapeutic inhibition of the PI3K/Akt pathway that induced HIF-1-a activity, re-established hypoxia-induced apoptosis [34, 44]. In contrast, Knowles et al*.* noted that *HIF-1-a* and *HIF-2-a* were not involved in mediating the increased apoptosis rate that they observed under hypoxia, as knockdown of either gene did not change apoptotic rates [36]. Interestingly, in diverse cancer types and non-cancerous tissues, it has been shown that hypoxia and HIFs can both trigger apoptosis and confer resistance to it [40, 45], which is in agreement with the described contradicting observations on the relationship between hypoxia, HIF-1-a, and apoptosis in EwS. As discussed in the section on proliferation, differentiating between finely adjusted hypoxia and HIF levels within experimental conditions as well as improvement and standardization of techniques could advance our understanding of EwS pathophysiology and possibly elucidate on the discrepancies in study findings up to date [19, 41].

### Migration and invasion

In contrast to the controversial effects of hypoxia and HIF-1-a on EwS cell proliferation and survival, its effects on cellular migration and invasion in EwS were more consistent across different studies. Most authors agreed on the increased migratory and invasive capacities of EwS cells that are exposed to hypoxia and on the fact that migration and invasion were mediated, at least in part, by HIF-1-a [29, 31, 33, 46–48]. Among the molecular mechanisms underlying invasiveness and migration under low oxygen tension, Krook et al*.* identified elevated expression of C-X-C motif chemokine receptor 4 (CXCR4) transcript and protein levels in EwS cells [48]. Additionally, several studies introduced the concept of activated SRC proto-oncogene (Src) and a feed-forward loop between Src and Tenascin C (TNC) that fostered matrix degradation and invadopodia formation in EwS under hypoxia [33, 47]. In fact, targeting of the Src/TNC axis inhibited EwS migration in vitro [47]. At the transcriptomic level, invasion gene signatures were upregulated when EwS cells were exposed to hypoxia [29]. HIF-1-a appeared crucial for mediating increased invasiveness and migration under hypoxia in EwS cells [31, 46]. This was evidenced by in vitro* HIF-1-a* knock down that reduced cellular invasion under hypoxia [46] but strikingly also under normoxia [31, 46]. However, for reasons that remain to be illuminated, Knowles et al*.* found that EwS cells migrated slower under hypoxia as compared to normoxia [36]. In this scenario, knockdown of *HIF-1-a* did not change the phenotype while knockdown of *HIF-2-a* partly reversed the hypoxic inhibition of migration [36].

### Colony formation and anchorage-independent growth

According to Aryee et al., hypoxia promoted anchorage-independent growth of EwS cell lines and marginally enhanced their clonogenicity [29]. Interestingly, EwS cells exposed to hypoxia could stimulate sphere formation of non-hypoxic EwS cells in their surrounding [35], which appeared to be mediated by HIF-1-a [35].

## The role of hypoxia and/or HIF-1-a activity in molecular signaling pathways in EwS

### HIF-1-a levels under normoxia and in response to hypoxia in EwS cells

A summary on these aspects is given in Table 1.

**Table 1:** HIF-1-a levels under normoxia and in response to hypoxia in EwS cells

Cell line	HIF-1-a detectable with western blot in normoxia	HIF-1-a detectable with western blot in hypoxia	Method to generate hypoxia	Reference
SK-N-MC	No	Yes	1%O2/ 5%CO2/ 95% N2 Spheroid growth	Aryee et al*.* 2010
	Yes	-	1%O2	Hameiri Grossmann et al*.* 2015
	Yes, very low expression	Yes, strong expression	0.1%O2/ 5%CO2/ balanceN2	Knowles et al*.* 2010
WE-68	No	Yes	1%O2/ 5%CO2/ 95% N2 Spheroid growth	Aryee et al*.* 2010
TC-252	No Yes	Yes	1%O2/ 5%CO2/ 95% N2 Spheroid growth	Aryee et al*.* 2010
TC-71	Yes	Yes	1%O2/ 5%CO2/ 95% N2 Spheroid growth	Aryee et al*.* 2010
	No	Yes	0.1%O2/ 5%CO2/ balanceN2	Knowles et al*.* 2010
RDES-1	Yes	-	1%O2	Hameiri Grossmann et al*.* 2015
	Yes, very low expression	Yes, strong expression	0.1%O2/ 5%CO2/ balanceN2	Knowles et al*.* 2010
MHH-ES-1	Yes	-	1%O2	Hameiri Grossmann et al*.* 2015
SK-ES-1	Yes	Yes, levels unchanged	1%O2	Hameiri Grossmann et al*.* 2015
	No	Yes	0.1%O2	Tilan et al*.* 2013
	No	Yes	1%O2/ 5%CO2/ 95% N2	Kling et al*.* 2020
	Yes, very low expression	Yes, strong expression	0.1%O2/ 5%CO2/ balanceN2	Knowles et al*.* 2010
A-673	No	Yes	0.5%O2	Kilic-Eren et al*.* 2013
	No	Yes	0.5%O2/ 5%CO2/ 95%N2	Kilic et al*.* 2007
	No	Yes	1%O2/ 5%CO2/ 95%N2	Kling et al*.* 2020
	No	Yes	0.1%O2/ 5%CO2 /balanceN2	Knowles et al*.* 2010
TC-32	No	Yes	1%O2	El Naggar et al*.* 2015
CHLA-10	Yes, very low expression	Yes, strong expression	1%O2	El Naggar et al*.* 2015
	No	Yes	1%O2/ 5%CO2/ 95%N2	El Naggar et al*.* 2019

### HIF-1-a and EWSR1::FLI1 interplay

Intra-tumor heterogeneity is a well-established tumor characteristic [49] that has been applied to the different expression levels of EWSR1::FLI1 that exist in EwS cells [50]. Apparently, EWSR1::FLI1 expression is dynamic within single cells, however the mechanism behind this fluctuation is not understood [50]. Similarly to EWSR1::FLI1, HIF-1-a expression has been shown to be heterogenous across EwS tumors and possibly also within a given EwS tumor [4, 29, 36]. Interestingly, in immunohistochemical analysis of EwS tumors and western blot of EwS cells, HIF-1-a mostly localized to the nucleus under normoxia [31, 36], contrasting with findings in skeletal muscle were HIF-1-a was located merely in the cytoplasm in normoxia [51]. Furthermore, HIF-1-a co-localized in some but not all tumor sections with areas of necrosis [36]. In summary, evidence exists that HIF-1-a, like EWSR1::FLI1, contributes to tumor heterogeneity in EwS.

Most interestingly, some reports indicated that HIF-1-a heterogeneity and EWSR1::FLI1 heterogeneity could be mechanistically linked to each other: Aynaud et al*.* showed that both very high and very low levels of EWSR1::FLI1 activity were associated with reduced EwS cell proliferation and upregulation of HIF-1-a target genes [30]. Furthermore, HIF-1-a directly induced EWSR1::FLI1 expression [29, 31]. In this context, we propose an alternative view, where HIF-1-a signaling an hypoxia may impact EWSR1::FLI1 expression levels independently. In fact, both, hypoxia-dependent and non-hypoxia-dependent HIF-1-a activation have been shown to induce EWSR1::FLI1 activity in EwS [29, 31]. However, for the hypoxia-mediated induction of EWSR1::FLI1, Aryee et al*.* showed that EWSR1::FLI1 protein levels were only transiently augmented and reverted to low expression levels within 24 h of hypoxia, while HIF-1-a expression seemed to be stably induced [29]. Based on these observations, we propose the following scenario for the interactions of hypoxia, HIF-1-a and EWSR1::FLI1 in EwS: Non-hypoxia-mediated HIF-1-a activity that induced EWSR1::FLI1 activity could describe the mechanism in the cell population characterized by high activity of both proteins, HIF-1-a and EWSR1::FLI1 [30, 31]. In contrast, hypoxia-mediated HIF-1-a activity that induces only transient elevation of EWSR1::FLI1 could be the mechanism describing the cell population that is characterized by high activity of HIF-1-a and low EWSR1::FLI1 activity [29, 30]. The non-proliferative state of this cell population fits in line with the notion that strong hypoxia is not compatible with proliferation in EwS [4]. Furthermore, these cells could be characterized by the two observations that EwS cells with low EWSR1::FLI [30, 50] and EwS cells exposed to hypoxia [29, 33, 47, 48] showed increased migratory and invasive potential. However, it is not clear why EwS cells with both high EWSR1::FLI1 and high HIF-1-a activity, are non-proliferative [30], and it could be of great interest to further characterize this cell population. One explanation could be that the high HIF-1-a activity itself prevents EwS proliferation, yet the exact influence of HIF-1-a on Ews proliferation is not clear to date (see section on proliferation). Of note, EWSR1::FLI1 induced by HIF-1-a via hypoxia was most probably upregulated on a posttranscriptional level [29], while EWSR1::FLI1 that was induced by HIF-1-a in normoxia via Ras signaling was upregulated via direct binding of HIF-1-a to the EWSR1::FLI1 promoter [31].

**Fig. 1:**
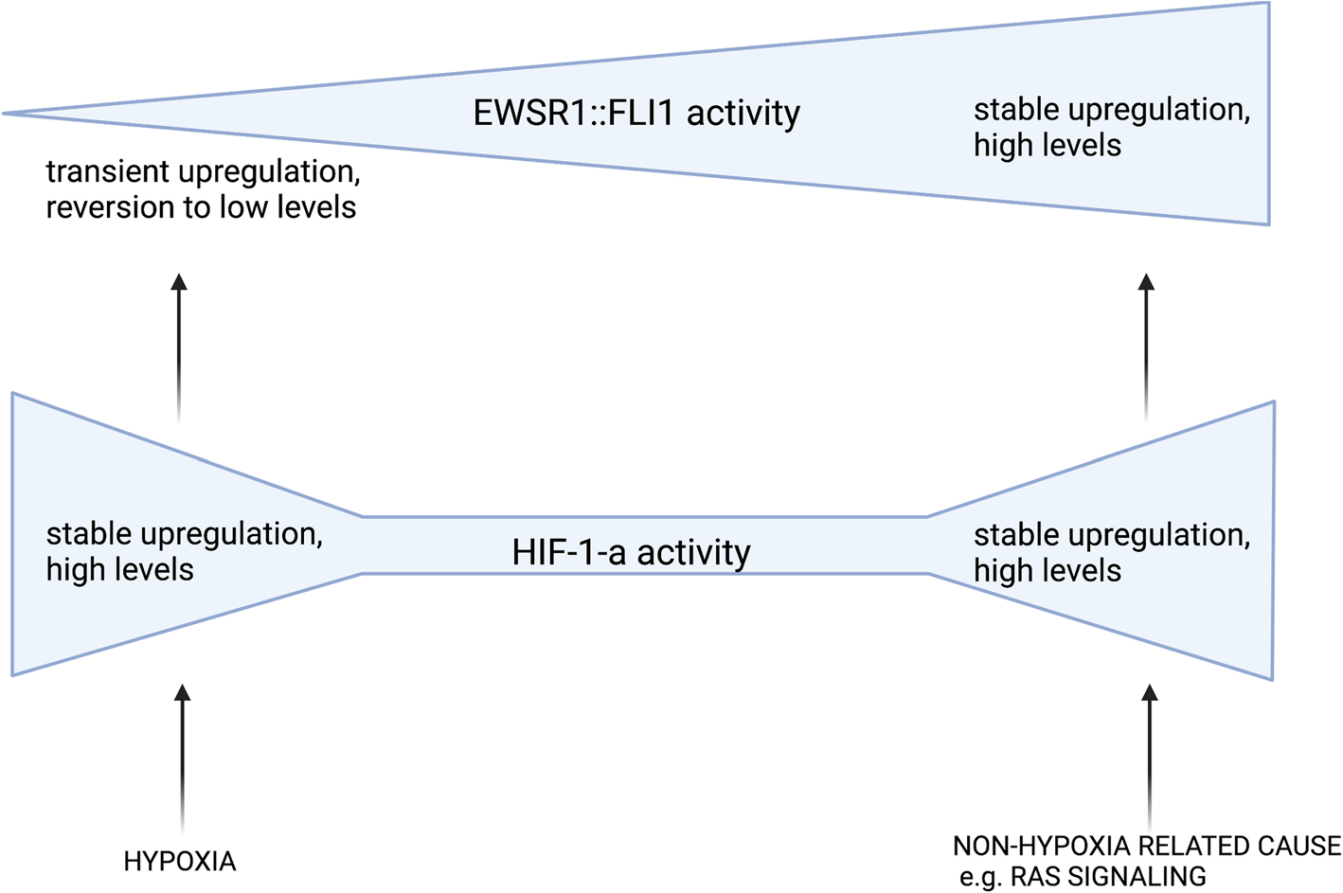
Hypoxia-related and non hypoxia-related upregulation of HIF-1-a might contribute independently to EWSR1::FLI1 regulation

Collectively, there is evidence that hypoxia and HIF-1-a are two key factors contributing to the dynamic regulation of EWSR1::FLI1 in EwS [29–31]. Based on the discussed reports we propose that both hypoxia, and HIF-1-a may contribute independently to the regulation of EWSR1::FLI1 (Fig. 1).

### Hypoxia and/or HIF-1-a and other molecular signaling pathways in EwS

In EwS, several molecules and signaling pathways have been identified to operate upstream of HIF-1-a and regulate its expression in normoxia as well as in hypoxia.

In terms of pathways that regulate HIF-1-a expression under normoxia, the Ras signaling cascade [12] could induce HIF-1-a in EwS [31]. Of note, no mutations were found that could explain the Ras activity in EwS cell lines [31]. Additionally, in normoxia, enchondral bone protein chondromodulin I (CHM1) regulated *HIF-1-a* levels in EwS by suppressing its expression [52]. CHM1 expression was induced by EWSR1::FLI1 and increased the potential of EwS cells for lung metastasis in vivo [52]. Furthermore, mutations in the Isocitrate dehydrogenase (IDH) enzyme may cause elevated cellular oxidative stress and stabilize HIF-1-a [53]. However, in a cohort of 61 patients diagnosed as EwS, *IDH1/2* mutations were rare [53], suggesting that *IDH1/2* mutations may play a minor role in HIF-1-a induction in EwS.

Some upstream regulators of HIF-1-a in EwS seem to operate both in normoxia and hypoxia: The Y-box binding protein 1 (YB-1) bound to the *HIF-1-a* five prime untranslated region (5’-UTR) and induced translation of its mRNA in normoxic and hypoxic conditions [46]. This emphasizes again that HIF-1-a levels and hypoxia are not exclusively interlinked. El Naggar et al*.* proposed that translational regulation of *HIF-1-a* via YB-1 might be a general mechanism for cancer cells to maintain elevated HIF-1-a levels independent from oxygen tension, while regulation of HIF-1-a via prolyl hydroxylase activity could represent a specific hypoxia-induced regulation mode [46]. Additionally, Src was involved in the mediation of a migratory and invasive response of EwS cells to hypoxia [32]. In diverse cancer types such as osteosarcoma, activated Src signaling was found to be a source for HIF-1-a stabilization in both, hypoxia and normoxia [54–58] and Src signaling has been suggested to play a role in sarcoma pathophysiology, including EwS [59, 60]. However, whether Src acts in EwS cells under hypoxia via the induction of HIF-1-a is still an open question, as well as whether Src is activated in EwS also under normoxia. Yet, the available data on Src signaling under hypoxia in EwS to date is compatible with these hypotheses [33, 47]. Despite evidence for a potential pro-tumorigenic role of Src [33, 47], its function in EwS remains controversial, since Zhou et al*.* showed that hyperactivity of Src inhibited EwS growth and migration in vitro and that EwS avoided Src hyperactivity via growth differentiation factor 6 (GDF6)/CD99 signaling [61]. Hence, further elucidation on the role of Src in EwS and its role in the context of hypoxia and HIFs is needed.

Finally, some molecular signaling pathways regulating HIF-1-a activity are operating mainly under hypoxia in EwS: Thus, phosphorylated ATM serine/threonine kinase (ATM) could possibly induce HIF-1-a expression under hypoxia in EwS as it co-localized in EwS spheroids with HIF-1-a staining [4]. In fact, ATM phosphorylated and stabilized HIF-1-a under hypoxia in mouse embryonic fibroblasts [62], yet the mechanistic connection between DDR enzymes and HIF-1-a in EwS remains to be elucidated. Additionally, the PI3K/Akt pathway seemed to be constitutively activated in EwS cell lines and essential for HIF-1-a induction and activity in hypoxic conditions [44]. Although PI3K/Akt signaling was also active in normoxia, no corresponding HIF-1-a expression was detected in EwS cell lines in normoxia [44]. Furthermore, and potentially downstream of HIF-1-a, CXCR4, which was induced via HIF-1-a under hypoxia in tumor entities such as gastric cancer [63, 64], was also induced by hypoxia in EwS [48]. However, the involvement of HIF-1-a in mediating CXCR4 signaling was not investigated. Intriguingly, Berghuis et al*.* did not detect a hypoxia dependent upregulation of CXCR4 on the cell surface of EwS cell lines [65]. Yet, it should be noted that both studies employed different cell lines except for TC-71, and none determined *HIF-1-a* mRNA levels [48, 65]. Mancarella et al*.* described insulin like growth factor 2 mRNA binding protein 3** (**IGF2BP3) as player in the CXCR4 signaling cascade [66] and concurred with Krook et al*.* on the relatedness of CXCR4 to hypoxia in EwS [48, 66]. Furthermore, the neuropeptide Y (NPY) pathway seemed to be active in EwS cells that were exposed to hypoxia [67, 68]. In fact, NPY seems to be a key molecule for the regulation of the EwS cell phenotype under hypoxia since it confers to them migration potential and cancer stem cell properties [68, 69]. Most importantly, the hypoxia-induced activation of the NPY/Y5 receptor (Y5R) pathway results via Rho-A over-activation in cytokinesis failure [69]. The originating polyploid EwS cells exhibit an aggressive phenotype with high chromosomal instability (CIN), bone invasiveness and chemotherapy resistance [69]. Finally, it should be noted that mutations in *TP53* and other genes were shown to influence the hypoxic phenotype of cancer cells [70]. Although there are no specific data on this aspect available for EwS, it is important to mention that TP53 mutations are commonly found in cell line models, but only in 5% of primary EwS tumors [24], wherefore studies on HIFs and hypoxia in EwS cell lines may be more presentative for this rare, but high-risk, patient population.

## Hypoxia and/or HIF-1-a activity and therapy and resistance in EwS

### Hypoxia and HIFs and prognostic markers in EwS

**Fig. 2:**
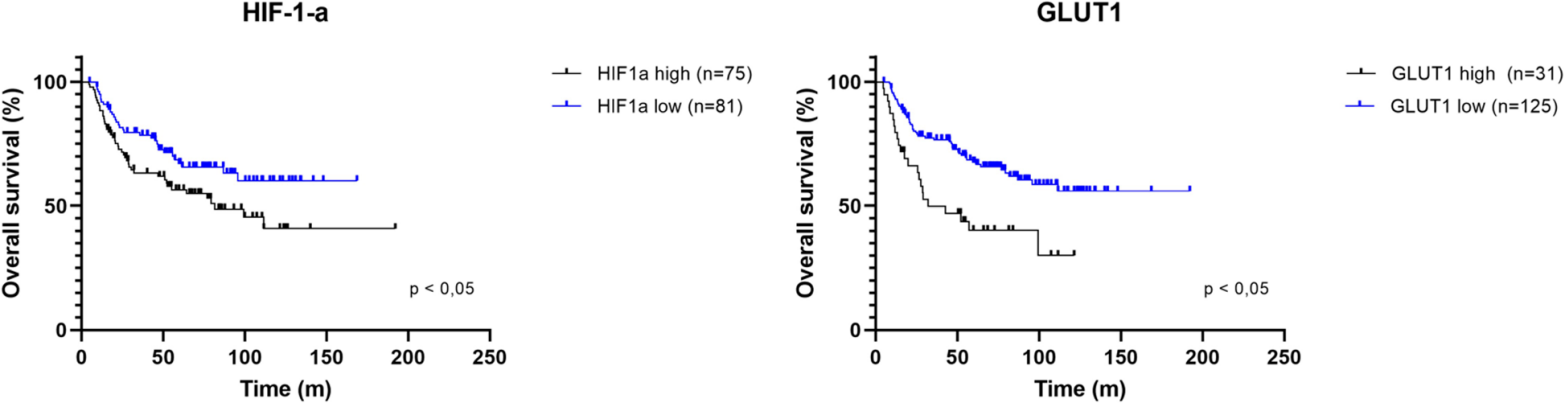
Elevated *HIF-1-a* and *GLUT1* expression correlates with worse overall survival in EwS patients. Kaplan–Meier survival analyses in 156 EwS patients based on *HIF-1-a* and *GLUT1* expression levels (cut-off defined as best percentile, log-rank test). Microarray data were retrieved from the Gene Expression Omnibus (accession codes: GSE63157, GSE34620, GSE12102, GSE17618) and normalized using Robust Multiarray Average (RMA) using custom brainarray chip-description files (v20). Batch effects were removed with ComBat. Tumor purity was assessed using the ESTIMATE algorithm. Only samples with a tumor purity > 60% corresponding to The Cancer Genome Atlas (TCGA) standard were included in survival analyses

Therapeutic options to target hypoxia in childhood cancers have been recently reviewed and the urgent need for prognostic markers to evaluate hypoxia in the pediatric setting has been highlighted [21]. Therefore, expression of HIF-1-a, HIF-2-a, and their downstream targets such as VEGF, GLUT1, carbonic anhydrase 9 (CA9), phosphoglycerate kinase 1 (PGK1), and lysyl oxidase (LOX) was evaluated and their association with prognosis and chemotherapy-response seemed to vary between pediatric cancer entities [21]. In this review, we evaluated the correlation of gene expression levels and survival in our cohort of 156 EwS patients and identified high *HIF-1-a* and *GLUT1* expression to be significantly associated with worse prognosis (Fig. 2), which was not observed for *PGK1*, *LOX*, *HIF-2-a*, *VEGF*, and *CA9* (not shown). Our results are in line with the notion of Bernauer et al*.*, that i) genes related to HIF signaling could serve as prognostic markers, and ii) the relationship between these genes and survival probably depends on the specific tumor type [21]. Regarding *HIF-1-a* expression at the mRNA level (*n* = 156), our data is in contrast with Knowles et al*.* who did not find a correlation between HIF-1-a expression and survival at the protein level in their EwS patient cohort [36]. Besides the possible difference between the mRNA and protein level, one additional explanation could be that the cohort of Knowles et al*.* cohort was perhaps too small (*n* = 56) to detect a significant difference in survival between HIF-1-a high and low expressing tumors. Most interestingly, HIF-1-a’s downstream effector *GLUT1* was associated to reduced survival in our cohort with very high significance, suggesting GLUT1 as potential biomarker for EwS prognosis (Fig. 2).

### Hypoxia and/or HIF-1-a activity and therapy in EwS

Targeting hypoxia in EwS treatment has been proposed since more than a decade [27, 28] and corresponding preclinical and clinical studies have been conducted. Preclinically, the Ras inhibitor salirasib reduced EwS growth and migration in vitro and in vivo [31]. Interestingly, salirasib also reduced HIF-1-a and EWSR1::FLI1 protein levels in vivo, suggesting its therapeutic potential in EwS treatment [31]. However, there are no clinical trials for salirasib in pediatric patients so far. Furthermore, melatonin induced hydroxylation and inactivation of HIF-1-a in EwS cell lines, leading to reduced aerobic glycolysis, increased reactive oxygen species (ROS) levels, and apoptosis [71]. Melatonin was well tolerated by pediatric patients in a dose-escalation study [72] and could be a promising candidate for further clinical investigation. Additionally, El Naggar et al*.* found that the class I histone deacetylase (HDAC) inhibitor MS-275, also named etinostat, inhibited YB-1 binding to target gene transcripts and constrained translation of stress-adaptive proteins, among them HIF-1-a [73]. Even though the report focused on NFE2 like bZIP transcription factor 2 (NFE2L2) as mechanistic explanation for the in vivo anti-tumor effects of MS-275 in EwS, the fact that MS-275 also decreased HIF-1-a translation should not be overlooked [73]. MS-275 was well tolerated in the pediatric setting, including EwS patients, and one study reported stable disease for one year under MS-275 treatment in a EwS patient [74, 75]. Further studies are needed to evaluate the therapeutic potential of MS-275 in EwS patients, potentially also in combination treatment. As mentioned above, the role of Src in hypoxic EwS cells is currently discussed and Bailey et al*.* demonstrated that dasatinib, a Src inhibitor, decreased EwS motility and invasion [33]. Yet, two caveats for the use of dasatinib are that i) it seemed not to inhibit proliferation rates in EwS cell lines and ii) the strong rebound effects that have been observed in vitro [33] which suggest that dasatinib should be combined with other drugs for EwS treatment. In line with this, single agent therapy with dasatinib was not efficient in EwS patients in a phase II study [76] and a phase I/II study testing the combination of dasatinib with additional chemotherapeutics in pediatric solid tumors is ongoing (NCT00788125). Furthermore, the CXCR4 signaling axis that is probably linked to hypoxia in EwS has been identified as therapeutic target to reduce EwS migration [48]. In a phase I/II study, the CXCR4 inhibitor plerixafor was well tolerated by pediatric patients, including patients with EwS [77]. However, plerixafor currently is only used as drug to mobilize hematopoietic stem cells from the bone marrow [77] and potential effects on EwS growth and metastasis have not been investigated clinically yet. Of note, bevacizumab, a monoclonal antibody against the HIF-1-a-downstream target VEGF, showed promising anti-tumor effects in combination treatment in EwS in two clinical studies [78, 79]. Additionally, studies in glioma cell lines and patient-derived colon cancer xenografts showed that irinotecan, which is known as DNA damaging anti-cancer agent, can downregulate *HIF-1-a* mRNA and protein levels [80–82]. This sheds light on the potential mode of action of irinotecan in EwS treatment, where it is already successfully applied [83–86]. Lastly, geldanamycin, which indirectly inhibits HIF-1-a, was tolerated in a phase I study by pediatric patients, including EwS patients [21, 87]. However, it remains unclear if this drug has anti-tumor efficiency in EwS and further studies are ongoing (NCT00093821). In summary, available preclinical and clinical data support the notion that targeting hypoxia, HIF-1-a, and their associated pathways represent a promising therapeutic strategy in EwS. In this context, drugs targeting hypoxia could be especially useful as an addition to the standard chemotherapeutics in EwS treatment [21, 78, 79]. However, phase II/III studies of hypoxia-targeting drugs in EwS are still missing, and further research in this field is urgently needed.

A summary of therapies in the context of hypoxia and HIFs in EwS is given in Table 2.

**Table 2:** Hypoxia and/or HIF-1-a activity and therapy in EwS

Drug	Mode of action	Preclinical data in EwS	Clinical data in pediatric EwS patients	Reference(s)
Salirasib	Ras inhibitor	In vitro and in vivo growth and migration inhibition Reduction of HIF-1-a levels in vivo	Not tested in pediatric patients	Hameiri-Grossman et al., 2015
Melatonin	Hydroxylation and inactivation of HIF-1-a	Induction of ROS and apoptosis in vitro, inhibition of glycolysis	Phase I study tested tolerability in pediatric patients with relapsed solid tumors, no EwS patients included	Sanchez-Sanchez et al*.*, 2015 Johnston et al*.*, 2019
Etinostat/ MS-275	Inhibiton of HIF-1-a translation via inhibition of YB-1 binding to HIF-1-a mRNA	Reduction of metastasis in vivo	Phase I studies show tolerability in pediatric patients including EwS patients	El-Naggar et al*.*, 2019 Bukowinski et al*.*, 2021 Gore et al*.*, 2008
Dasatinib	Src inhibitor	In vitro inhibition of migration and invasion, no inhibition of proliferation	Phase II study showed no efficiency as single agent in EwS Phase I/II study on combinational treatment in children with solid tumors ongoing	Bailey et al*.*, 2016 Schuetze et al*.*, 2016 NCT00788125
Plerixafor	CXCR4 inhibitor	In vitro inhibition of migration and invasion	Phase I/II study demonstrated tolerability in pediatric patients including EwS patients	Krook et al*.*, 2014 Morland et al., 2020
Bevacizumab	VEGF monoclonal antibody	*-*	Tolerability and responses in EwS patients treated with a combination treatment including Bevacizumab	Kuo et al*.*, 2017 Wagner et al*.*, 2013
Irinotecan	DNA damaging agent	No data in EwS available Reduced *HIF-1-a* levels in glioma and colon cancer	Tolerability and efficacy of combined regimens including irinotecan in advanced EwS patients	Casey et al*.*, 2009 Kurucu et al*.*, 2015 Salah et al*.*, 2021 Xu et al*.*, 2021
Geldanamycin	Indirect inhibition of HIF-1-a	-	Phase I study demonstrated tolerability in pediatric patients including EwS patients	Bagatell et al*.*, 2007 Bernauer et al*.*, 2021 NCT00093821
Imatinib	Tyrosine-kinase inhibitor	In vitro reduction of HIF-1-a protein levels that were induced under hypoxia (± metformin) In vivo reduction of metastasis in combination treatment with metformin	Phase II studies demonstrated tolerability but no efficacy of imatinib as single agent therapy in relapsed EwS patients	Nan et al*.*, 2020 Chugh et al*.*, 2009 Bond et al*.*, 2008

### Hypoxia and/or HIF-1-a activity and therapy resistance in EwS

Hypoxia-induced drug resistance is a well-established concept [21, 88] that has been explored also in EwS. For example, Batra et al*.* found that hypoxia impairs fenretinide (4-HPR) therapy in EwS through the upregulation of acid ceramidase, which fosters the conversion of pro-apoptotic ceramid species into the pro-survival molecule sphingosine-1-phosphate [89]. Through combination with safingol, the anti-tumor effect of 4-HPR under hypoxic in vitro conditions could be reestablished [89]. A clinical phase I study defined the maximal tolerated dosage of oral 4-HPR in pediatric patients with high-risk solid tumors, which included five EwS patients [90]. 4-HPR was well tolerated and stabilized tumor growth in one of the five EwS patients [90]. The suggested combination of 4-HPR with safingol [89] could probably improve therapy effectiveness, but still needs to be investigated. As another example, metformin promisingly reduced proliferation of EwS cells and sensitized them to chemotherapeutics in vitro, such as vincristine and doxorubicin [91]. However, in vivo experiments did not show any reduction in tumor proliferation through metformin, neither as single agent therapy nor in combination with other chemotherapeutics [91]. In fact, hypoxia, which was present in vivo but not in vitro, counteracted the anti-proliferative effects of metformin that were observed in vitro [91]. Accordingly, hypoxia had a substantial impact on EwS therapy options implying that more physiological-like cell culture methods in the field of EwS and drug discovery are urgently needed. Most interestingly, Nan et al*.* found that imatinib could reverse hypoxia-induced resistance of EwS cells to metformin, most probably via inhibition of HIF-1-a activity [39]. Hence, concomitant application of metformin and imatinib reduced EwS proliferation and metastasis in vitro and in vivo and suggested this combination as a powerful new therapeutic approach in EwS [39]. However, imatinib as single agent therapy was not effective in EwS patients in two phase II studies conducted so far [92, 93]. Currently, metformin as addition to chemotherapy is tested for children with solid tumors in a phase I study (NCT01528046) as well as its potential use for maintenance therapy of children and adults with bone sarcoma (NCT04758000). Furthermore, Kilic et al*.* found GLUT1 expression downstream of HIF-1-a as well as the PI3K/Akt pathway that contributed to resistance of EwS cell line A-673 to chemotherapeutics such as doxorubicin, vincristine, and actinomycin D under hypoxia [34, 44]. Of note, the A-673 cell line is a p53 deficient cell line, but the same hypoxia-induced drug resistance was also observed in the p53 wildtype rhabdomyosarcoma cell line A204 [34, 44]. Further evidence for the involvement of the PI3K/Akt pathway in hypoxia-induced drug resistance in EwS is that the tyrosine kinase inhibitor imatinib reduced HIF-1-a levels in EwS cells and thereby reversed hypoxia-induced resistance to metformin [39]. Moreover, Magwere et al*.* demonstrated that hypoxia-induced drug resistance in EwS was heterogenous across different chemotherapeutics and cell lines, thus adding complexity to the topic [37]. Of note, glutathione (GSH) levels in response to hypoxia were also heterogenous across EwS cell lines, indicating that the GSH antioxidant system is probably not ideally suited for therapeutic targeting of hypoxia-induced drug resistance in EwS [37]. Finally, a recent study uncovered the NPY/Y5R-RhOA axis as potential mechanism of hypoxia-induced chemoresistance in EwS [69]. The authors demonstrated that Y5R inhibition successfully reduced hypoxia induced EwS disease recurrence in bones in vivo and thereby strongly underlined the rationale for targeting the hypoxic cell population within a EwS tumor [69].

## Hypoxia and/or HIF-1-a activity and EwS metabolism

One of the hallmarks of cancer is the reprograming of the energy metabolism, to fuel its uncontrolled cell growth [94]. In EwS, EWSR1::FLI1 mediated upregulation of enzymes involved in serine-glycine biosynthesis [95–97] and glucose metabolism [98, 99], as well as increased expression of glutamine transporters [96]. Furthermore, EWSR1::FLI1 inhibited the breakdown of tryptophan in the kynurenine pathway thus hindering aryl hydrocarbon receptor (AHR) signaling [100]. HIF-1-a is known to play an important role as a regulator of cancer metabolism, mainly through shifting it from an oxidative to a glycolytic form [14, 101]. In cancer cells reciprocal upregulation exists between HIF-1-a and glycolysis [101, 102]. Furthermore, HIF-1-a-mediated induction of glycolytic enzymes can arise independently from hypoxia, possibly explaining the Warburg effect [14]. Along these lines, aerobic glycolysis, characteristic of the Warburg effect, was found in EwS cell lines but not in chondrosarcoma or non-malignant cell lines [71, 98, 103]. Moreover, a direct link between HIF-1-a and aerobic glycolysis in EwS cells [71] as well as a direct link between hypoxia and GLUT-1 expression and glucose uptake [34, 36] have been described. Additionally, when EwS cell lines were exposed to low glucose levels, a significant increase in HIF-1-a and HIF-2-a expression was found [36], illustrating again the potential for hypoxia-independent upregulation of HIF-1-a in EwS cells. Nevertheless, it is not yet elucidated if and how EWSR1::FLI1 and HIF-1-a act together to change EwS metabolism and how this may be potentially exploited for targeted therapies.

## Hypoxia and/or HIF-1-a activity and acidosis in EwS

Increased glycolysis leads to intracellular and extracellular acidification and thus contributes to tumor acidosis, which was shown to be true in bone sarcomas [104]. Accordingly, HIF-1-a signaling seemed to be crucial in these events, regardless of whether HIF-1-a activation happened due to hypoxia or not [104]. Most interestingly, DiPompo et al*.* suggested that tumor acidosis could reciprocally influence HIF-1-a levels in bone sarcomas, for example via pH-dependent nucleolar sequestration of von Hippel-Lindau tumor suppressor (VHL) or nuclear factor kappa B (NF-kB) signaling, and for the later one evidence has already been found in osteosarcoma cells [104]. Avnet et al*.* found that EwS cells employed the V-ATPase proton pump to maintain pH homeostasis during tumor acidosis, suggesting V-ATPase as potential target for EwS treatment [103]. All in all, little is known about the role that hypoxia and HIF-1-a signaling play in EwS tumor acidosis, however the discussed studies suggest that further research in this field could open new therapeutic opportunities.

## Hypoxia and/or HIF-1-activity and EwS metastasis

The presence of metastasis at diagnosis is the strongest predictor for poor outcome in EwS [24]. Metastasis is a complex process selecting for highly aggressive tumor cells through sequential steps including exit and migration from the primary tumor, penetration of blood vessels, survival through circulation, and adaptation to distant organs, where cells must adjust to tissue-specific microenvironmental signals [105]. In this context, hypoxia and HIF-dependent signaling are emerging as key microenvironmental promoters of metastasis [69, 106]. In fact, the amount of tumor ischemia was linked to increased metastasis in EwS patients [32]. High HIF-1-a expression was shown to correlate with sarcoma metastasis in in situ and in vivo murine models; potentially due to HIF-1-a-mediated orchestration of collagen-associated tumor cell transportation and penetration into the vasculature [107]. In EwS, *HIF-1-a* transcriptional activation was mediated by YB-1 and lead to increased invasive and metastatic potential in vivo [46]. Interestingly, targeting YB-1 by increasing acetylation using the class I HDAC inhibitor MS-275 proved to enhance oxidative stress and decrease metastatic potential in vivo [73]. Additionally, hypoxia contributed to EwS metastasis by transforming NPY from a cell death mediator into a growth- and migration-promoting factor through selective regulation of its Y2R/Y5R receptors [68, 108]. In this context, EwS patients with higher systemic NPY levels in serum showed worse malignancy features [109]. Specifically, EwS tumors that were subjected to hypoxia developed a high capacity to metastasize to the bone niche and Y5R inhibitors reduced bone invasiveness and bone metastasis in EwS in vivo [69]. Finally, the above discussed combination of metformin with imatinib for EwS treatment inhibited the formation of metastases in an in vivo murine model [39]. In summary, these studies show that several mechanisms of metastasis in EwS are mediated by hypoxia and HIF-dependent signaling, which opens new inroads for therapeutic targeting of tumor progression.

## Hypoxia and/or HIF-1-a activity and EwS vasculature

EwS employs three different strategies to promote the expansion of the vasculature: i) angiogenesis, ii) vasculogenesis and iii) tumor cell vascular mimicry [110].

Angiogenesis is the sprouting of new vessels from pre-existing ones and develops in response to tumor hypoxia [111]. EwS cells replying to hypoxia promoted the release of angiogenic factors form the surrounding stroma and additionally expressed themselves VEGF, CXCR4, and fibroblast growth factors (FGFs) to bring on the angiogenic switch [112]. One key regulator in this process was the zinc finger WT1 transcription factor (WT1) [113]: WT1 was upregulated in response to hypoxia, directly induced transcription of VEGF and thus assisted in angiogenic activities and tube formation of endothelial cells in EwS [113]. Vasculogenesis is the process in which bone marrow (BM) cells, endothelial cells, and pericytes/vascular smooth muscle cells (vSMC) organize to form the tumor vascular network [114]. A downregulation of delta like canonical Notch ligand 4 (DLL4) was correlated with reduced pericytes/vSMCs covering of the vessels, making them leak and increasing EwS hypoxia [115]. Furthermore, repressor element 1-silencing transcription factor (REST), was identified to be a key regulator of EwS vessel proficiency. Intriguingly, low expression of this EWSR1::FLI1 target gene impaired EwS vessel morphology and increased tumor hypoxia [116, 117]. Lastly, the ability of tumor cells to form microvascular channels in hypoxic microenvironments is called ‘vascular mimicry’ [118]. HIF-1-a was highly expressed by EwS cells around blood lakes and could drive vascular mimicry in those tumor cells [119]. Additionally, EwS cells surrounding blood lakes also expressed Y2R, implying involvement of Y2R and NPY in EwS vascular mimicry [68]. In summary, hypoxia and HIF-1-a have been found to promote vascular expansion in EwS throughout different mechanisms, highlighting their potential therapeutic value in EwS treatment.

## Hypoxia and/or HIF-1-a activity and EwS endochondral ossification

EwS mainly arises in bones [24] and hypoxia plays an important role during bone development, specifically the process of endochondral ossification (ECO) [120, 121]. In fact, hypertrophic chondrocytes must overcome hypoxia to enable bone maturation, which they do via HIF-1-a signaling and induction of VEGF [120, 121]. This presence of angiogenic factors in the microenvironment could ultimately create a well-suiting soil for Ewing sarcomagenesis [25, 111]. Furthermore, evidence exists for crosstalk of EWSR1::FLI1 with diverse transcription factors of bone development, such as induction of SRY-box transcription factor 6 (SOX6) through EWSR1::FLI1 [122], the direct binding of EWSR1::FLI1 to RUNX family transcription factor 2 (RUNX2) [123, 124], and the indirect influence of EWSR1::FLI1 on SRY-box transcription factor 9 (SOX9) regulation [125]. On a similar note, association of SOX6 and SOX9 expression with hypoxia/HIF-1-a has been found in the context of bone formation [126, 127]. Based on these findings we suggest ECO-related hypoxia/HIF-1-a signaling as potential determinants in EwS pathogenesis, yet more research in this field is needed. Of note, the bone niche and the associated hypoxic conditions as key factors influencing EwS pathophysiology have already been discussed [25–27]. Accordingly, hypoxia as an integral part of the bone microenvironment attracted EwS cells that had previously been subjected to hypoxia to metastasize specifically to the bone niche in vivo but not to other compartments [69]. Furthermore, inhibition of the Y5R precisely reduced bone metastasis in vivo but not metastasis in other locations [69]. This underlines the important role of the TME and indicates intra-tumoral heterogeneity among EwS tumor cells [69, 108]. Interestingly, hypoxia was key to generate EWSR1::FLI1-driven EwS models from human mesenchymal stem cells derived from a EwS patient [128]. Finally, hypoxia and/or HIFs play a major role for osteoclast stimulation during bone resorption [129] and extensive osteolytic bone destruction has been called a principal characteristic of EwS [27].

## Hypoxia and/or HIF-1-a activity and exosomes in EwS

Investigating the nature and role of tumor exosomes in sarcoma development has recently gained more attention [130]. Kling et al*.* addressed the effect of hypoxia on EwS exosomes and found that the cargo of hypoxic EwS exosomes contained elevated microRNA 210 levels in comparison to EwS exosomes secreted by normoxic cells [35]. In fact, these hypoxic EwS exosomes enhanced survival and sphere formation capacity in normoxic EwS cells when co-cultured [35]. Consequently, hypoxic EwS cells seem to be able to influence the non-hypoxic cells within the same tumor, adding to the complexity of the hypoxic EwS TME.

## Hypoxia and/or HIF-1-a activity and chromosomal instability in EwS

CIN as continuing errors in chromosomal segregation during successive cell divisions [131] is a common phenomenon across cancer entities including EwS [69, 131]. The resulting genomic instability promotes tumor cell adaptation to harsh environmental conditions and probably confers aggressiveness to EwS tumors [69]. Most interestingly, hypoxia causes CIN and aneuploidy in EwS cells via the NPY/Y5R-RhoA-axis [69]. This might ultimately increase EwS disease recurrence and metastatic potential [69]. Of note, EwS cells that were exposed to hypoxia keep their tendency for mitotic segregation errors and CIN even upon reoxygenation, indicating that EwS cells keep a cellular memory of having been exposed to hypoxia [69].

## HIF-1-b and EwS

HIF-1-b, also known as ARNT, is not only the dimerization partner of HIF-1-a, but also of additional transcription factors, including AHR, single minded proteins (SIM), and c-Jun proteins [17, 132]. Upregulation of ARNT/HIF-1-b has been associated with multiple types of cancer [133–135]. In fact, *ARNT* locates to chromosome region 1q21, which is found amplified in different tumors, including EwS [136–139]. Regarding the role of ARNT in EwS, one study demonstrated that ARNT could contribute to proliferation, antiapoptotic capacities and angiogenesis of EwS cells [140]. Of note, signaling of ARNTs dimerization partner AHR has been shown to contribute to tumor progression and low survival in chronic lymphocytic leukemia and glioma patients [141]. In EwS, interactions of EWSR1::FLI1 with the AHR signaling pathway have been proposed [100]. However, it is not clear which role ARNT plays in this context, yet these findings could suggest ARNT as potential target for EwS therapy.

## Conclusion

This review summarizes emerging evidence that hypoxia and HIF signaling are involved in EwS pathophysiology in multiple ways, e.g., in migration and metastasis, metabolism, and formation of vasculature, highlighting the importance of studying them. Based on previous reports, we introduced the concept of viewing hypoxia and HIFs independently from each other when looking at molecular interactions of HIF-1-a and EWSR1::FLI1, yet this hypothesis needs to be further validated. Additionally, have shown in our EwS patient cohort that expression of *HIF-1-a* and downstream targets is associated with worse prognosis, underlying the clinical relevance of hypoxia and HIFs in EwS. Lastly, preclinical, and clinical studies give proof that therapeutic targeting of hypoxia, HIFs, and associated pathways could improve the outcome of EwS patients. This is specifically true for combination therapies [21, 78, 79] implying that rational treatment combinations connecting anti-HIF/hypoxia agents with other therapeutics are likely to produce the strongest improvement, which is especially relevant for EwS patients with metastatic or relapsed disease [24].

## Data Availability

Microarray data were retrieved from the Gene Expression Omnibus (accession codes: GSE63157, GSE34620, GSE12102, GSE17618).

## Abbreviations

EwS: Ewing sarcoma
HIF-1-a: Hypoxia inducible factor 1 subunit alpha
TME: Tumor microenvironment
HIF: Hypoxia inducible factor
HRE: Hypoxia response element
VEGF: Vascular endothelial growth factor
GLUT1: Solute carrier family 2 member 1 (SLC2A1, better known as GLUT-1)
PI3K: Phosphatidylinositol-4,5-bisphosphate 3-kinase
Akt: AKT serine/threonine kinase 1
MAPK: Mitogen-activated protein kinase
PTEN: Phosphatase and tensin homolog protein
HIF-1-b: Hypoxia inducible factor 1 subunit beta
ARNT: Aryl hydrocarbon receptor nuclear translocator
EWSR1: Ewing sarcoma breakpoint region 1 protein
FLI1: Friend leukaemia integration 1 transcription factor
DDR: DNA damage repair
CXCR4: C-X-C motif chemokine receptor 4
CIN: Chromosomal instability
Src: SRC proto-oncogene
TNC: Tenascin C
CHM1: Chondromodulin I
IDH: Isocitrate dehydrogenase
YB-1: Y-box binding protein 1
5’-UTR: Five prime untranslated region
GDF6: Growth differentiation factor 6
ATM: ATM serine/threonine kinase
IGF2BP3: Insulin like growth factor 2 mRNA binding protein 3
NPY: Neuropeptide Y
CA9: Carbonic anhydrase 9
PGK1: Phosphoglycerate kinase 1
LOX: Lysyl oxidase
ROS: Reactive oxygen species
HDAC: Histone deacetylase
NFE2L2: NFE2 like bZIP transcription factor 2
4-HPR: Fenretinide
GSH: Glutathione
AHR: Aryl hydrocarbon receptor
VHL: Von Hippel-Lindau tumor suppressor
NF-kB: Nuclear factor kappa B
FGF: Fibroblast growth factor
WT1: WT1 transcription factor
BM: Bone marrow
vSMC: Vascular smooth muscle cell
DLL4: Delta like canonical Notch ligand 4
REST: Repressor element 1-silencing transcription factor
ECO: Endochondral ossification
SOX6: SRY-box transcription factor 6
RUNX2: RUNX family transcription factor 2
SOX9: SRY-box transcription factor 9
SIM: Single minded protein

## References

[1] Balkwill FR, Capasso M, Hagemann T (2012). The tumor microenvironment at a glance. J Cell Sci.

[2] Bregenzer ME (2019). Integrated cancer tissue engineering models for precision medicine. PLoS ONE.

[3] Hanahan D (2022). Hallmarks of Cancer: New Dimensions. Cancer Discov.

[4] Riffle S, Pandey RN, Albert M, Hegde RS (2017). Linking hypoxia, DNA damage and proliferation in multicellular tumor spheroids. BMC Cancer.

[5] Petrova V, Annicchiarico-Petruzzelli M, Melino G, Amelio I (2018). The hypoxic tumour microenvironment. Oncogenesis.

[6] Pouysségur J, Dayan F, Mazure NM (2006). Hypoxia signalling in cancer and approaches to enforce tumour regression. Nature.

[7] Wang GL, Semenza GL (1993). Characterization of hypoxia-inducible factor 1 and regulation of DNA binding activity by hypoxia. J Biol Chem.

[8] Hammond EM (2014). The Meaning, Measurement and Modification of Hypoxia in the Laboratory and the Clinic. Clin Oncol.

[9] Semenza GL, Roth PH, Fang HM, Wang GL (1994). Transcriptional regulation of genes encoding glycolytic enzymes by hypoxia-inducible factor 1. J Biol Chem.

[10] Esteban MA (2006). Regulation of E-cadherin Expression by VHL and Hypoxia-Inducible Factor. Cancer Res.

[11] Jiang Y-G (2007). Role of Wnt/β-catenin signaling pathway in epithelial-mesenchymal transition of human prostate cancer induced by hypoxia-inducible factor-1α: Prostate cancer undergoes EMT via Wnt. Int J Urol.

[12] Bárdos JI, Ashcroft M (2004). Hypoxia-inducible factor-1 and oncogenic signalling: Review articles. BioEssays.

[13] Semenza GL (2002). Signal transduction to hypoxia-inducible factor 1. Biochem Pharmacol.

[14] Hayashi Y, Yokota A, Harada H, Huang G (2019). Hypoxia/pseudohypoxia-mediated activation of hypoxia-inducible factor-1α in cancer. Cancer Sci.

[15] Masoud GN, Li W (2015). HIF-1α pathway: role, regulation and intervention for cancer therapy. Acta Pharm Sin B.

[16] Mandl M, Depping R (2014). Hypoxia-Inducible Aryl Hydrocarbon Receptor Nuclear Translocator (ARNT) (HIF-1β): Is It a Rare Exception?. Mol Med.

[17] Wolff M, Jelkmann W, Dunst J, Depping R (2013). The Aryl Hydrocarbon Receptor Nuclear Translocator (ARNT/HIF-1β) is Influenced by Hypoxia and Hypoxia-Mimetics. Cell Physiol Biochem.

[18] Wouters BG, Koritzinsky M (2008). Hypoxia signalling through mTOR and the unfolded protein response in cancer. Nat Rev Cancer.

[19] McKeown SR (2014). Defining normoxia, physoxia and hypoxia in tumours—implications for treatment response. Br J Radiol.

[20] Bao MH-R, Wong CC-L (2021). Hypoxia, Metabolic Reprogramming, and Drug Resistance in Liver Cancer. Cells.

[21] Bernauer C (2021). Hypoxia and its therapeutic possibilities in paediatric cancers. Br J Cancer.

[22] Pierrevelcin M (2020). Focus on Hypoxia-Related Pathways in Pediatric Osteosarcomas and Their Druggability. Cells.

[23] Zhang Y, et al. Hypoxia in Breast Cancer—Scientific Translation to Therapeutic and Diagnostic Clinical Applications. Front Oncol. 2021;11:652266. 10.3389/fonc.2021.652266 PMC7991906 33777815

[24] Grünewald TGP (2018). Ewing sarcoma. Nat Rev Dis Primer.

[25] Ehnman M, Chaabane W, Haglund F, Tsagkozis P (2019). The Tumor Microenvironment of Pediatric Sarcoma: Mesenchymal Mechanisms Regulating Cell Migration and Metastasis. Curr Oncol Rep.

[26] Molina ER, Chim LK, Barrios S, Ludwig JA, Mikos AG (2020). Modeling the Tumor Microenvironment and Pathogenic Signaling in Bone Sarcoma. Tissue Eng Part B Rev.

[27] Redini F, Heymann D. Bone Tumor Environment as a Potential Therapeutic Target in Ewing Sarcoma. Front Oncol. 2015;5:279. 10.3389/fonc.2015.00279 PMC4688361 26779435

[28] Zeng W, Wan R, Zheng Y, Singh SR, Wei Y (2011). Hypoxia, stem cells and bone tumor. Cancer Lett.

[29] Aryee DNT (2010). Hypoxia Modulates EWS-FLI1 Transcriptional Signature and Enhances the Malignant Properties of Ewing’s Sarcoma Cells In vitro. Cancer Res.

[30] Aynaud M-M (2020). Transcriptional Programs Define Intratumoral Heterogeneity of Ewing Sarcoma at Single-Cell Resolution. Cell Rep.

[31] Hameiri-Grossman M (2015). The association between let-7, RAS and HIF-1α in Ewing Sarcoma tumor growth. Oncotarget.

[32] Dunst J, Ahrens S, Paulussen M, Burdach S, Jürgens H (2001). Prognostic Impact of Tumor Perfusion in MR-Imaging Studies in Ewing Tumors: Strahlenther. Onkol.

[33] Bailey KM, Airik M, Krook MA, Pedersen EA, Lawlor ER (2016). Micro-Environmental Stress Induces Src-Dependent Activation of Invadopodia and Cell Migration in Ewing Sarcoma. Neoplasia N Y N.

[34] Kilic M, Kasperczyk H, Fulda S, Debatin K-M (2007). Role of hypoxia inducible factor-1 alpha in modulation of apoptosis resistance. Oncogene.

[35] Kling MJ (2020). Exosomes secreted under hypoxia enhance stemness in Ewing’s sarcoma through miR-210 delivery. Oncotarget.

[36] Knowles HJ, Schaefer K-L, Dirksen U, Athanasou NA (2010). Hypoxia and hypoglycaemia in Ewing’s sarcoma and osteosarcoma: regulation and phenotypic effects of Hypoxia-Inducible Factor. BMC Cancer.

[37] Magwere T, Burchill SA (2011). Heterogeneous role of the glutathione antioxidant system in modulating the response of ESFT to fenretinide in normoxia and hypoxia. PLoS ONE.

[38] Hoogsteen IJ (2005). Colocalization of Carbonic Anhydrase 9 Expression and Cell Proliferation in Human Head and Neck Squamous Cell Carcinoma. Clin Cancer Res.

[39] Nan X (2020). Imatinib revives the therapeutic potential of metformin on ewing sarcoma by attenuating tumor hypoxic response and inhibiting convergent signaling pathways. Cancer Lett.

[40] Greijer AE (2004). The role of hypoxia inducible factor 1 (HIF-1) in hypoxia induced apoptosis. J Clin Pathol.

[41] Pavlacky J, Polak J (2020). Technical Feasibility and Physiological Relevance of Hypoxic Cell Culture Models. Front Endocrinol.

[42] Place TL, Domann FE, Case AJ (2017). Limitations of oxygen delivery to cells in culture: An underappreciated problem in basic and translational research. Free Radical Biol Med.

[43] Ryland KE (2015). Polycomb-dependent repression of the potassium channel-encoding gene KCNA5 promotes cancer cell survival under conditions of stress. Oncogene.

[44] Kilic-Eren M, Boylu T, Tabor V (2013). Targeting PI3K/Akt represses Hypoxia inducible factor-1α activation and sensitizes Rhabdomyosarcoma and Ewing’s sarcoma cells for apoptosis. Cancer Cell Int.

[45] Bacon A, Harris A (2004). Hypoxia-inducible factors and hypoxic cell death in tumour physiology. Ann Med.

[46] El-Naggar AM (2015). Translational Activation of HIF1α by YB-1 Promotes Sarcoma Metastasis. Cancer Cell.

[47] Hawkins AG (2019). Microenvironmental Factors Drive Tenascin C and Src Cooperation to Promote Invadopodia Formation in Ewing Sarcoma. Neoplasia.

[48] Krook MA (2014). Stress-induced CXCR4 promotes migration and invasion of ewing sarcoma. Mol Cancer Res MCR.

[49] Meacham CE, Morrison SJ (2013). Tumour heterogeneity and cancer cell plasticity. Nature.

[50] Franzetti G-A (2017). Cell-to-cell heterogeneity of EWSR1-FLI1 activity determines proliferation/migration choices in Ewing sarcoma cells. Oncogene.

[51] Kubis HP, Hanke N, Scheibe RJ, Gros G (2005). Accumulation and nuclear import of HIF1 alpha during high and low oxygen concentration in skeletal muscle cells in primary culture. Biochim Biophys Acta BBA Mol Cell Res..

[52] von Heyking K (2017). The endochondral bone protein CHM1 sustains an undifferentiated, invasive phenotype, promoting lung metastasis in Ewing sarcoma. Mol Oncol.

[53] Na KY (2015). IDH Mutation Analysis in Ewing Sarcoma Family Tumors. J Pathol Transl Med.

[54] Karni R, Dor Y, Keshet E, Meyuhas O, Levitzki A (2002). Activated pp60c-Src Leads to Elevated Hypoxia-inducible Factor (HIF)-1α Expression under Normoxia. J Biol Chem.

[55] Lee H-Y (2011). Src activates HIF-1α not through direct phosphorylation of HIF-1α-specific prolyl-4 hydroxylase 2 but through activation of the NADPH oxidase/Rac pathway. Carcinogenesis.

[56] Sudhagar S, Sathya S, Lakshmi BS (2011). Rapid non-genomic signalling by 17β-oestradiol through c-Src involves mTOR-dependent expression of HIF-1α in breast cancer cells. Br J Cancer.

[57] Takacova M, et al. Src induces expression of carbonic anhydrase IX via hypoxia-inducible factor. Oncol Rep. 2010;23(3):869-74. 20127031

[58] Vettori A (2017). Glucocorticoids promote Von Hippel Lindau degradation and Hif-1α stabilization. Proc Natl Acad Sci.

[59] Chen Q (2015). The importance of Src signaling in sarcoma. Oncol Lett.

[60] Indovina P (2017). SRC Family Kinase Inhibition in Ewing Sarcoma Cells Induces p38 MAP Kinase-Mediated Cytotoxicity and Reduces Cell Migration: SFK INHIBITION IN EWING SARCOMA CELLS. J Cell Physiol.

[61] Zhou F (2020). GDF6-CD99 Signaling Regulates Src and Ewing Sarcoma Growth. Cell Rep.

[62] Cam H, Easton JB, High A, Houghton PJ (2010). mTORC1 Signaling under Hypoxic Conditions Is Controlled by ATM-Dependent Phosphorylation of HIF-1α. Mol Cell.

[63] Korbecki J (2021). The Effect of Hypoxia on the Expression of CXC Chemokines and CXC Chemokine Receptors—A Review of Literature. Int J Mol Sci.

[64] Oh YS (2012). Hypoxia induces CXCR4 expression and biological activity in gastric cancer cells through activation of hypoxia-inducible factor-1α. Oncol Rep.

[65] Berghuis D (2012). The CXCR4-CXCL12 axis in Ewing sarcoma: promotion of tumor growth rather than metastatic disease. Clin Sarcoma Res.

[66] Mancarella C (2020). Insulin-Like Growth Factor 2 mRNA-Binding Protein 3 Modulates Aggressiveness of Ewing Sarcoma by Regulating the CD164-CXCR4 Axis. Front Oncol.

[67] Tilan J, Kitlinska J (2016). Neuropeptide Y (NPY) in tumor growth and progression: Lessons learned from pediatric oncology. Neuropeptides.

[68] Tilan JU (2013). Hypoxia shifts activity of neuropeptide Y in Ewing sarcoma from growth-inhibitory to growth-promoting effects. Oncotarget.

[69] Lu C (2022). Hypoxia-activated neuropeptide Y/Y5 receptor/RhoA pathway triggers chromosomal instability and bone metastasis in Ewing sarcoma. Nat Commun.

[70] Zhang C (2021). The Interplay Between Tumor Suppressor p53 and Hypoxia Signaling Pathways in Cancer. Front Cell Dev Biol.

[71] Sanchez-Sanchez AM (2015). Melatonin Cytotoxicity Is Associated to Warburg Effect Inhibition in Ewing Sarcoma Cells. PLoS ONE.

[72] Johnston DL (2019). Phase I dose-finding study for melatonin in pediatric oncology patients with relapsed solid tumors. Pediatr Blood Cancer.

[73] El-Naggar AM (2019). Class I HDAC inhibitors enhance YB-1 acetylation and oxidative stress to block sarcoma metastasis. EMBO Rep.

[74] Bukowinski A, et al. A phase 1 study of entinostat in children and adolescents with recurrent or refractory solid tumors, including CNS tumors: Trial ADVL1513, Pediatric Early Phase‐Clinical Trial Network (PEP‐CTN). Pediatr Blood Cancer. 2021;68(4):e28892. 10.1002/pbc.28892 PMC9176707 33438318

[75] Gore L (2008). A Phase I and Pharmacokinetic Study of the Oral Histone Deacetylase Inhibitor, MS-275, in Patients with Refractory Solid Tumors and Lymphomas. Clin Cancer Res.

[76] Schuetze SM (2016). SARC009: Phase 2 study of dasatinib in patients with previously treated, high-grade, advanced sarcoma: Dasatinib in Patients With Sarcoma. Cancer.

[77] Morland B (2020). Plerixafor combined with standard regimens for hematopoietic stem cell mobilization in pediatric patients with solid tumors eligible for autologous transplants: two-arm phase I/II study (MOZAIC). Bone Marrow Transplant.

[78] Kuo C (2017). Docetaxel, bevacizumab, and gemcitabine for very high risk sarcomas in adolescents and young adults: A single-center experience: Kuo et al. Pediatr Blood Cancer..

[79] Wagner L (2013). Pilot study of vincristine, oral irinotecan, and temozolomide (VOIT regimen) combined with bevacizumab in pediatric patients with recurrent solid tumors or brain tumors: VOIT With Bevacizumab for Recurrent Pediatric Tumors. Pediatr Blood Cancer.

[80] Guérin E (2012). In Vivo Topoisomerase I Inhibition Attenuates the Expression of Hypoxia-Inducible Factor 1α Target Genes and Decreases Tumor Angiogenesis. Mol Med.

[81] Kamiyama H, Takano S, Tsuboi K, Matsumura A (2005). Anti-angiogenic effects of SN38 (active metabolite of irinotecan): inhibition of hypoxia-inducible factor 1 alpha (HIF-1?)/vascular endothelial growth factor (VEGF) expression of glioma and growth of endothelial cells. J Cancer Res Clin Oncol.

[82] Sapra P (2011). Potent and sustained inhibition of HIF-1α and downstream genes by a polyethyleneglycol-SN38 conjugate, EZN-2208, results in anti-angiogenic effects. Angiogenesis.

[83] Casey DA (2009). Irinotecan and temozolomide for Ewing sarcoma: The Memorial Sloan-Kettering experience. Pediatr Blood Cancer.

[84] Kurucu N, Sari N, Ilhan IE (2015). Irinotecan and Temozolamide Treatment for Relapsed Ewing Sarcoma: A Single-Center Experience and Review of the Literature. Pediatr Hematol Oncol.

[85] Salah S (2021). Irinotecan and temozolomide chemotherapy in paediatric and adult populations with relapsed Ewing Sarcoma. Clin Transl Oncol.

[86] Xu J (2021). Anlotinib, Vincristine, and Irinotecan for Advanced Ewing Sarcoma After Failure of Standard Multimodal Therapy: A Two-Cohort Phase Ib/II Trial. The Oncologist.

[87] Bagatell R (2007). Phase I Pharmacokinetic and Pharmacodynamic Study of 17- N -Allylamino-17-Demethoxygeldanamycin in Pediatric Patients with Recurrent or Refractory Solid Tumors: A Pediatric Oncology Experimental Therapeutics Investigators Consortium Study. Clin Cancer Res.

[88] Jing X (2019). Role of hypoxia in cancer therapy by regulating the tumor microenvironment. Mol Cancer.

[89] Batra S, Reynolds CP, Maurer BJ (2004). Fenretinide cytotoxicity for Ewing’s sarcoma and primitive neuroectodermal tumor cell lines is decreased by hypoxia and synergistically enhanced by ceramide modulators. Cancer Res.

[90] Villablanca JG (2006). Phase I Trial of Oral Fenretinide in Children With High-Risk Solid Tumors: A Report From the Children’s Oncology Group (CCG 09709). J Clin Oncol.

[91] Garofalo C (2013). Metformin as an Adjuvant Drug against Pediatric Sarcomas: Hypoxia Limits Therapeutic Effects of the Drug. PLoS ONE.

[92] Bond M (2008). A phase II study of imatinib mesylate in children with refractory or relapsed solid tumors: A Children’s Oncology Group study. Pediatr Blood Cancer.

[93] Chugh R (2009). Phase II Multicenter Trial of Imatinib in 10 Histologic Subtypes of Sarcoma Using a Bayesian Hierarchical Statistical Model. J Clin Oncol.

[94] Hanahan D, Weinberg RA (2011). Hallmarks of Cancer: The Next Generation. Cell.

[95] Issaq SH (2020). EWS-FLI1–regulated Serine Synthesis and Exogenous Serine are Necessary for Ewing Sarcoma Cellular Proliferation and Tumor Growth. Mol Cancer Ther.

[96] Sen N (2018). EWS-FLI1 reprograms the metabolism of Ewing sarcoma cells via positive regulation of glutamine import and serine-glycine biosynthesis. Mol Carcinog.

[97] Tanner JM (2017). EWS/FLI is a Master Regulator of Metabolic Reprogramming in Ewing Sarcoma. Mol Cancer Res.

[98] Dasgupta A (2017). Metabolic modulation of Ewing sarcoma cells inhibits tumor growth and stem cell properties. Oncotarget.

[99] Yeung C (2019). Targeting Glycolysis through Inhibition of Lactate Dehydrogenase Impairs Tumor Growth in Preclinical Models of Ewing Sarcoma. Cancer Res.

[100] Mutz CN (2016). EWS-FLI1 impairs aryl hydrocarbon receptor activation by blocking tryptophan breakdown via the kynurenine pathway. FEBS Lett.

[101] Semenza GL (2010). HIF-1: upstream and downstream of cancer metabolism. Curr Opin Genet Dev.

[102] Lu H, Forbes RA, Verma A (2002). Hypoxia-inducible Factor 1 Activation by Aerobic Glycolysis Implicates the Warburg Effect in Carcinogenesis. J Biol Chem.

[103] Avnet S (2013). V-ATPase is a candidate therapeutic target for Ewing sarcoma. Biochim Biophys Acta.

[104] Di Pompo G, Cortini M, Baldini N, Avnet S (2021). Acid Microenvironment in Bone Sarcomas. Cancers.

[105] Weiss F, Lauffenburger D, Friedl P (2022). Towards targeting of shared mechanisms of cancer metastasis and therapy resistance. Nat Rev Cancer.

[106] Rankin EB, Giaccia AJ (2016). Hypoxic control of metastasis. Science.

[107] Eisinger-Mathason TSK (2013). Hypoxia-Dependent Modification of Collagen Networks Promotes Sarcoma Metastasis. Cancer Discov.

[108] Hong S-H (2015). High neuropeptide Y release associates with Ewing sarcoma bone dissemination - in vivo model of site-specific metastases. Oncotarget.

[109] Tilan JU (2015). Systemic levels of neuropeptide Y and dipeptidyl peptidase activity in patients with Ewing sarcoma-Associations with tumor phenotype and survival: NPY in Patients With Ewing Sarcoma. Cancer.

[110] DuBois SG, Marina N, Glade-Bender J (2010). Angiogenesis and vascular targeting in Ewing sarcoma: A review of preclinical and clinical data. Cancer.

[111] Llombart-Bosch A, López-Guerrero JA, Batalla CC, Suari AR, Peydró-Olaya A. Structural Basis of Tumoral Angiogenesis. in New Trends in Cancer for the 21stCentury (eds. Llombart-Bosch, A. & Felipo, V.) vol. 532 69–89 (Springer US, 2003). 10.1007/978-1-4615-0081-0_8 12908551

[112] Giner F (2019). Chemokine Expression Is Involved in the Vascular Neogenesis of Ewing Sarcoma: A Preliminary Analysis of the Early Stages of Angiogenesis in a Xenograft Model. Pediatr Dev Pathol.

[113] McCarty G, Awad O, Loeb DM (2011). WT1 protein directly regulates expression of vascular endothelial growth factor and is a mediator of tumor response to hypoxia. J Biol Chem.

[114] Stewart KS, Kleinerman ES (2011). Tumor Vessel Development and Expansion in Ewing’s Sarcoma: A Review of the Vasculogenesis Process and Clinical Trials with Vascular-Targeting Agents. Sarcoma.

[115] Schadler KL, Zweidler-McKay PA, Guan H, Kleinerman ES (2010). Delta-like ligand 4 plays a critical role in pericyte/vascular smooth muscle cell formation during vasculogenesis and tumor vessel expansion in Ewing’s sarcoma. Clin Cancer Res Off J Am Assoc Cancer Res..

[116] Zhou Z, Yu L, Kleinerman ES (2014). EWS-FLI-1 regulates the neuronal repressor gene REST, which controls Ewing sarcoma growth and vascular morphology. Cancer.

[117] Zhou Z, Yang Y, Wang F, Kleinerman ES (2020). Neuronal Repressor REST Controls Ewing Sarcoma Growth and Metastasis by Affecting Vascular Pericyte Coverage and Vessel Perfusion. Cancers.

[118] Wei X (2021). Mechanisms of vasculogenic mimicry in hypoxic tumor microenvironments. Mol Cancer.

[119] van der Schaft DWJ (2005). Tumor cell plasticity in Ewing sarcoma, an alternative circulatory system stimulated by hypoxia. Cancer Res.

[120] Mackie EJ, Ahmed YA, Tatarczuch L, Chen K-S, Mirams M (2008). Endochondral ossification: How cartilage is converted into bone in the developing skeleton. Int J Biochem Cell Biol.

[121] Schipani E, Mangiavini L, Merceron C. HIF-1α and growth plate development: what we really know. BoneKEy Rep. 2015;4:730. 10.1038/bonekey.2015.99 PMC4549921 26331009

[122] Marchetto A (2020). Oncogenic hijacking of a developmental transcription factor evokes vulnerability toward oxidative stress in Ewing sarcoma. Nat Commun.

[123] Li X, McGee-Lawrence ME, Decker M, Westendorf JJ (2010). The Ewing’s sarcoma fusion protein, EWS-FLI, binds Runx2 and blocks osteoblast differentiation. J Cell Biochem.

[124] Marturano-Kruik A (2018). Biomechanical regulation of drug sensitivity in an engineered model of human tumor. Biomaterials.

[125] Merkes C (2015). Ewing Sarcoma Ewsa Protein Regulates Chondrogenesis of Meckel’s Cartilage through Modulation of Sox9 in Zebrafish. PLoS ONE.

[126] Amarilio R (2007). HIF1α regulation of Sox9 is necessary to maintain differentiation of hypoxic prechondrogenic cells during early skeletogenesis. Development.

[127] Khan WS, Adesida AB, Tew SR, Lowe ET, Hardingham TE (2010). Bone marrow-derived mesenchymal stem cells express the pericyte marker 3G5 in culture and show enhanced chondrogenesis in hypoxic conditions: BMSs EXPRESS PERICYTE MARKERS IN CULTURE. J Orthop Res.

[128] Sole A (2021). Unraveling Ewing Sarcoma Tumorigenesis Originating from Patient-Derived Mesenchymal Stem Cells. Cancer Res.

[129] Knowles H. Hypoxic regulation of osteoclast differentiation and bone resorption activity. Hypoxia. 2015;73. 10.2147/HP.S95960. 10.2147/HP.S95960 PMC5045091 27774484

[130] Chicón-Bosch M, Tirado OM (2020). Exosomes in Bone Sarcomas: Key Players in Metastasis. Cells.

[131] Bakhoum SF, Cantley LC (2018). The Multifaceted Role of Chromosomal Instability in Cancer and Its Microenvironment. Cell.

[132] Huang W-C (2010). Involvement of aryl hydrocarbon receptor nuclear translocator in EGF-induced c-Jun/Sp1-mediated gene expression. Cell Mol Life Sci.

[133] Gardella KA (2016). Aryl hydrocarbon receptor nuclear translocator (ARNT) isoforms control lymphoid cancer cell proliferation through differentially regulating tumor suppressor p53 activity. Oncotarget.

[134] Huang C-R (2015). Down-regulation of ARNT promotes cancer metastasis by activating the fibronectin/integrin β1/FAK axis. Oncotarget.

[135] Zhao Y, Han F, Zhang X, Zhou C, Huang D (2020). Aryl hydrocarbon receptor nuclear translocator promotes the proliferation and invasion of clear cell renal cell carcinoma cells potentially by affecting the glycolytic pathway. Oncol Lett.

[136] Mosakhani N (2012). An integrated analysis of miRNA and gene copy numbers in xenografts of Ewing’s sarcoma. J Exp Clin Cancer Res.

[137] Tarkkanen M (1999). Clinical Correlations of Genetic Changes by Comparative Genomic Hybridization in Ewing Sarcoma and Related Tumors. Cancer Genet Cytogenet.

[138] Udayakumar AM, et al. Cytogenetic characterization of Ewing tumors using fine needle aspiration samples: a 10-year experience and review of the literature. Cancer Genet Cytogenet. 2001;127(1):42-8. 10.1016/s0165-4608(00)00417-9 11408064

[139] Watkins TBK (2020). Pervasive chromosomal instability and karyotype order in tumour evolution. Nature.

[140] Chen J (2018). MiR-107 suppresses cell proliferation and tube formation of Ewing sarcoma cells partly by targeting HIF-1β. Hum Cell.

[141] Sadik A (2020). IL4I1 Is a Metabolic Immune Checkpoint that Activates the AHR and Promotes Tumor Progression. Cell.

